# Cellular metabolism and oxidative stress as a possible determinant for longevity in small breed and large breed dogs

**DOI:** 10.1371/journal.pone.0195832

**Published:** 2018-04-25

**Authors:** Ana Gabriela Jimenez, Josh Winward, Ursula Beattie, William Cipolli

**Affiliations:** 1 Colgate University, Department of Biology, Hamilton, New York, United States of America; 2 Colgate University, Department of Mathematics, Hamilton, New York, United States of America; RIKEN Advanced Science Institute, JAPAN

## Abstract

Among species, larger animals tend to live longer than smaller ones, however, the opposite seems to be true for dogs—smaller dogs tend to live significantly longer than larger dogs across all breeds. We were interested in the mechanism that may allow for small breeds to age more slowly compared with large breeds in the context of cellular metabolism and oxidative stress. Primary dermal fibroblasts from small and large breed dogs were grown in culture. We measured basal oxygen consumption (OCR), proton leak, and glycolysis using a Seahorse XF96 oxygen flux analyzer. Additionally, we measured rates of reactive species (RS) production, reduced glutathione (GSH) content, mitochondrial content, lipid peroxidation (LPO) damage and DNA (8-OHdg) damage. Our data suggests that as dogs of both size classes age, proton leak is significantly higher in older dogs, regardless of size class. We found that all aspects of glycolysis were significantly higher in larger breeds compared with smaller breeds. We found significant differences between age classes in GSH concentration, and a negative correlation between DNA damage in puppies and mean breed lifespan. Interestingly, RS production showed no differences across size and age class. Thus, large breed dogs may have higher glycolytic rates, and DNA damage, suggesting a potential mechanism for their decreased lifespan compared with small breed dogs.

## Introduction

Aging is defined as a decrease in physiological function and fitness with age [1], which is progressive, endogenously derived, and irreversible for the organism. Due to accumulation of deleterious traits in its forward progress [2], every aspect of an organism’s phenotype is modified during aging [3]. Though, the influential role of the rate of energy expenditure and longevity across species has been discarded by gerontologists [4], mitochondria are still central to potential aging mechanisms. During aging, mitochondria become larger and less numerous, mitochondrial respiration activity decreases and damage to mitochondrial DNA increases [5]. Additionally, mitochondrial RS production increase during aging, often leading to parallel increases in amounts of oxidative damage [6]. These traits suggest that mitochondria have an important role in the aging process, though other hallmarks of aging such as genomic instability, telomere attrition, loss of proteostatis, stem cell exhaustion and altered intercellular communication do not seem to be fully related to mitochondria. Similarly, increases in glycolysis have often been implicated in age-related pathologies [7]. The literature points to several aging theories, including, but not limited to, oxidative stress, inflammation, telomere shortening, mitochondrial dysfunction and misfolded proteins [8]. Here, we chose to focus on cellular metabolic rate changes as well as its closely linked effects of oxidative stress on the relationship between body size and lifespan in dogs.

Dog sizesrange from the 2 kg Chihuahua to the 90 kg Great Dane, a 44-fold difference in body size [9], and smaller dogs tend to live significantly longer than larger dogs across all breeds [9–12]. With longer lifespans, smaller dogs also demonstrate whole-animal evolutionary differences by having smaller litter sizes and shorter growth trajectories compared with large breed dogs [13].

Cellular parameters may be involved in determining maximal lifespan in dogs. Primary fibroblasts have been established as a useful model system for many animals because of their ease of use in tissue culture and abundance in the body [14]. In fact, cellular resistance and aging rates have been tested using primary fibroblasts in eight different species of mammals ranging in body masses from 0.1 to 450 kg [15]. Cellular resistance is a corollary of the free radical theory of aging, where longer-lived, larger mammals should have higher resistance to chemical insults compared with shorter-lived, smaller mammals. Kapahi et al. [15] found that primary fibroblasts cells isolated from large, longer-lived mammals resisted chemical stress better than primary fibroblasts isolated from smaller, shorter-lived mammals suggesting that primary fibroblasts are an excellent model for the whole-animal phenotype.

Dog size influences metabolic rate which, in turn, may influence cellular phenotypes and mitochondrial function [16]. Mitochondrial function is associated with aging because it is the main source of energy in cells, and its byproducts, reactive species (RS), are the main culprits of oxidative damage within cells. As a byproduct of normal oxidative phosphorylation, errant electrons form free-radicals such as O_2_^-^, OH^-^, or H_2_O_2_, which can attack DNA, proteins, and lipids, causing impairment of function and ultimately cell death, if the damage cannot be repaired. At low levels, RS are essential in gene regulation, cell signaling, and apoptosis [17, 18] but at high levels, RS production can potentially overwhelm the antioxidant capacity of the cell and exert oxidative stress changing gene expression, and causing structural damage [17, 18]. Cells inherently contain molecules to combat damage from RS production, broadly termed the antioxidant system, which includes enzymatic antioxidants, such as glutathione peroxidase (GPx), superoxide dismutase (SOD) and catalase (CAT). These molecules function by catalyzing the oxidation of less biologically insulting molecules. Other antioxidant molecules such as vitamin E and C, act as chain-breaking antioxidants, which can scavenge for RS, remove them once they are formed, and further halt propagation of peroxidation [19]. Despite having a thwarting antioxidant system, oxidative damage accrues during times of stress and/or across animals’ lifespans. Two of the most insidious types of oxidative damage include damage to lipids in the form of lipid peroxidation (LPO), where an errant electron propagates across membranes, altering lipid composition and decreasing cellular function [20]. The other type of damage is directly to DNA molecules, accumulating mutations that can lead to diseases like cancer [21, 22]. The “oxidative stress” theory of aging states that aging is not a genetically programmed phenomenon, but it happens because of the deleterious damage of oxidative stress on the genetic machinery. This theory has now become the current accepted theory of biological aging according to gerontologists because it successfully links the balance between the accumulation of cellular damage and pro-oxidant concentration over time, thus giving rise to the process of aging, however, it is not the sole theory that encapsulates aging [23, 24]. Longer developmental trajectories seem to be associated, at the cell level, with increases in RS production, thus, larger dog breeds could amass higher cell turnover. Although cell turnover is required for continual growth, it may lead to mutations that are not repaired or eliminated leading to early senescence or disease [25]. Therefore, larger dog breeds could be burdened with increases in oxidative damage, such as lipid peroxidation damage or increases in DNA damage, for prolonged periods of time during early life, leading to higher rates of diseases, and hence early mortality [25–28]. Additionally, whereas adult dog weight ranges two orders of magnitude, the range in weights in new born puppies is significantly less variable (9), indicating that differences in lifespan may be accrued during growth.

That “large dogs die young” has been a contested statement in the dog literature. Mortality data have been used to attest that larger breeds “die young,” an approach prone to a number of unavoidable biases [27, 28], and others have used mathematical models of the ratio between birth mass and adult mass to yield similar conclusions [29], highlighting that larger breeds may have faster cellular damage accumulation leading to significantly shorter lives compared with smaller breeds. Empirical data demonstrating a physiological mechanism that underlies these differences has yet to be elucidated [30]. In this study, we grew primary fibroblast cells isolated from medically discarded tissue belonging to large and small breed puppies and large and small breed euthanized aged dogs. We compared cellular oxygen consumption, glycolysis and oxidative stress across these 4 groups to describe a potential physiological mechanism of the disparity of aging rates in dogs. To our knowledge, this is the first time empirical data has been collected to support that accumulation of oxidative damage and differences in cellular metabolism in larger dogs may be the culprit of their shorter lifespans.

## Methods

### Establishment of fibroblast cell lines

We isolated primary fibroblast cells from puppies and senior dogs of two size classes. The small breed size class was composed of breeds with an adult body mass of 15 kg or less. The large breed size class included breeds or mixes with an adult body mass of 20 kg or more. Puppy samples were samples obtained from routine tail docks, ear clips and dewclaw removals performed at veterinarian offices in Central New York and Michigan (listed in acknowledgements) (Table 1). Samples from older dogs were obtained via ear clips from euthanized dogs (Table 2). The samples were placed in cold transfer media (Dulbecco’s modified Eagle medium [DMEM], with 4.5 g/L glucose, sodium pyruvate, and 4 mM L-glutamine supplemented with 10% heat-inactivated fetal bovine serum, and antibiotics [100 U/mL pen/strep], containing 10 mM HEPES) and transferred to Colgate University. To isolate primary fibroblast cells, skin samples were sterilized in 70% ethanol, and 20% bleach. Once any fat and bone was removed, skin was minced and incubated in sterile 0.5% Collagenase Type 2 (Worthington Chemicals, Cat. No. LS004176) overnight in an atmosphere of 37 °C, 5% CO_2_/O_2_. After incubation, the Collagenase mixture was filtered through a 20 μm sterile mesh, and centrifuged at 1000 rpm for 5 min. The resulting supernatant was removed, and the pellet was resuspended with 7 mL of mammal media (Dulbecco’s modified Eagle medium [DMEM], with 4500 mg/L glucose, sodium pyruvate, and 4 mM L-glutamine supplemented with 10% heat-inactivated fetal bovine serum, and antibiotics [100 U/mL pen/strep]). Cells were grown in culture flasks at 37 °C in an atmosphere of 5% O_2_/CO_2_. When cells reached 90% confluence, they were trypsinized (0.25%) and cryopreserved at 10^6^ cells/mL in DMEM supplemented with 40% fetal bovine serum and dimethylsulfoxide (DMSO) at a final concentration of 10%. We stored cells in liquid N_2_ prior to any experiments. All measurements were conducted using cells at passage 2 (P2). All cell lines were thawed, resuspended and allowed 5 days to recover from freezing before oxygen consumption rate (OCR), extracellular acidification rate (ECAR) experiments and oxidative stress experiments. All the procedures within this study were approved by the Colgate University's Institutional Care and Use Committee's IACUC.

**Table 1:** Dog breeds separated by size class, number of litters and total puppies per breed included in our study, as well as average body mass per litter and average age at collection of sample.

Breed	Size class	Number of litters	Total N	Average body mass per litter (Kg)	Average age at collection (days)
Airedale terrier	Large	1	7	0.34	5
Australian Shepherd	Large	1	1	0.56	6
Boxer	Large	2	12	0.37	3.5
Boxer pitbull cross	Large	1	5	0.11	4
Bracco Italiano	Large	1	1	0.56	5
Cane corso	Large	2	5	5.45	36
Cane corso rottweiler cross	Large	1	2	N/A	4
Doberman	Large	6	15	3.24	53
Doberman mix	Large	1	1	5.89	53
Dogo Argentino	Large	1	1	18.6	91
English Mastiff	Large	1	3	N/A	4
German short-haired pointer	Large	1	10	0.52	2
German wire-haired pointer	Large	1	8	0.51	2
Great Dane	Large	1	12	0.85	5
Labradoodle	Large	1	10	0.51	3
Labrador retriever	Large	1	8	0.47	1
Large munsterlander	Large	1	10	0.37	2
Old English sheepdog	Large	2	17	0.34	2.5
Pitbull Mix	Large	1	1	4.5	56
Rottweiler	Large	3	14	0.45	4.5
Standard poodle	Large	2	19	0.45	4
Cavalier King Charles Spaniel	Small	1	5	0.24	<1
Chihuahua	Small	1	1	0.15	<1
Corgi	Small	1	6	N/A	6
Havanases	Small	1	2	0.26	7
Miniature poodle	Small	1	3	0.17	1
Miniature schnauzer	Small	1	2	0.29	4
Pomeranian	Small	1	2	0.17	<1
Soft coated wheaten terrier	Small	4	14	0.37	4.5
Toy poodle	Small	3	18	0.2	4.5
Yorkshire terrier chihuahua mix	Small	1	1	3.13	90
Yorkshire terrier	Small	4	16	0.08	4.5

**Table 2:** Dog breeds, size class and samples size of older euthanized dogs included in our study, as well as age at collection and reason for euthanasia.

Breed	Size class	Total N	Average body mass (Kg)	Average age at collection (years)	Reason for euthanasia
Alaskan Malamute cross	Large	1	26.31	15	Cancer
American Bulldog	Large	1	42.18	12	Cancer
Austrailian Shepherd	Large	1	25.67	8.5	Heart disease
Bassett Hound	Large	1	26.31	12	Digestive
Beagle	Large	1	20.87	12	Cancer
Border collie cross	Large	1	27.67	10	Perianal fistula
Boxer	Large	3	29.48	11.6	Cancer
Boxer mix	Large	1	18.14	11	Liver failure
Chocolate labrador retriever	Large	3	35.38	10	Cancer
Doberman	Large	1	34.02	9	Cancer
German Shepherd	Large	1	35.38	11	Kidney disease
German Shepherd cross	Large	4	33.11	12	Old age, Cancer
Golden retriever	Large	1	34.02	13	Cancer
Great dane	Large	1	47.17	6	Renal and heart disease
Husky	Large	1	27.22	14	Cough
Husky cross	Large	1	29.03	14.4	Old age
Lab cross	Large	2	32.41	9.75	Cancer
Pitbull	Large	1	29.48	8	pyometra
Pitbull cross	Large	1	27.22	11	Injuries
Rottweiler	Large	2	39.91	9.5	Hing leg paralysis, cancer
Shepherd cross	Large	2	33.79	9	Chronic anorexia, N/A
Yellow labrador retriever	Large	1	28.21	13	N/A
Bichon frise	Small	1	4.63	15	Old age
Cavalier/Bichon cross	Small	1	10.43	14	N/A
Chihuahua cross	Small	1	4.99	16	Old age
Corgi	Small	1	10.89	15	Cancer
Golden doodle	Small	1	7.89	10	Kidney disease
Jack Russell Terrier	Small	2	7.65	14	Old age, N/A
Miniature pinscher	Small	1	4.54	15	Old age
Miniature Schnauzer	Small	2	7.65	13.16	Cancer, Old age
Pomeranian	Small	1	4.54	13	Old age/spinal injury
Pomeranian/Chihuahua cross	Small	1	5.9	13	Heart Failure
Pug	Small	2	8.66	12.22	Rear leg weakness, fecal incontinence, jaundice
Shih Tzu	Small	1	5.26	14	Congestive heart failure
Shih Tzu cross	Small	1	7.26	13	Seizures
Terrier cross	Small	1	6.26	14	Old age
Yorkshire terrier	Small	1	9.07	15	Old age

### Metabolic profiles

A Seahorse XF-96 Extracellular flux analyzer was used to measure the rate of O_2_ uptake and glycolysis rates in primary dermal fibroblast cells from all dogs. Assays were performed prior to experiments to determine optimal cell seeding density, and optimal concentrations of each compound. We seeded 20,000 cells per well in duplicate per individual into XF-96 cell culture plates and allowed cells to attach overnight.

### Oxygen consumption rates (OCR)

OCR was determined using XF-96 FluxPaks from Agilent Technologies. We measured OCRs after cells were equilibrated to running media for 1 h, which contained 10 mM glucose, 1 mM sodium pyruvate and 2 mM glutamine, pH = 7.4. Baseline measurements of OCRs were made three times prior to injecting a final well concentration of 2 μM oligomycin, which inhibits ATP synthesis by blocking the proton channel of the Fo portion of the ATP synthase. This baseline is used to distinguish the percentage of O_2_ consumption devoted to ATP synthesis and O_2_ consumption required to overcome the natural proton leak across the inner mitochondrial membrane plus any non-mitochondrial O_2_ consumption. We then injected a final well concentration of 0.125 μM carbonyl cyanide-*4*-(trifluoromethoxy)phenylhydrazone (FCCP), an uncoupling agent that disrupts ATP synthesis by collapsing the proton gradient across the mitochondrial membrane leading to uncoupled consumption of energy and O_2_ without generating ATP, providing a theoretical maximal respiratory rate. Finally, we injected a final well concentration of 0.5 μM Antimycin A, a Complex III inhibitor and rotenone, a Complex I inhibitor. This combination stops mitochondrial respiration and enables non-mitochondrial respiration to be evaluated [31–33].

After measurements were completed, we used a Countess II FL cell counter to count the actual final concentration of cells in each well and normalized all rates to 20,000 cells. For each OCR parameter, we followed equations supplied by [34].

### Extra cellular acidification rate (ECAR)

ECAR values were measured in units of mpH, which is the change in pH in the media surrounding the cells due to proton flux in glycolysis. Measurements of ECAR were performed after the cells were equilibrated to running media for 1 h. Running media contained no glucose and 2 mM L-glutamine in all experiments, pH = 7.4. Baseline rates were measured three times prior to any injections. We, first, injected a final well concentration of 10 mM glucose into media surrounding cells, which provides a measure of glycolytic rate, and then injected a final well concentration of 2 μM Oligomycin, giving an estimate of glycolytic capacity in cells. Finally, we injected a final well concentration of 50 mM 2-DG, a glucose analog that inhibits glycolysis, providing an estimate of non-glycolytic acidification [33]. After measurements were completed, we used a Countess II FL cell counter to count the actual final concentration of cells in each well and normalized all rates to a total of 20,000 cells. For each ECAR parameter, we followed the equations supplied by [34].

### Oxidative stress profiles

For oxidative stress measurements, cells were seeded at 10,000 cells per well and allowed to attach for 24 h prior to any experiments. We used Thermo Scientific^™^ Nunc^™^ MicroWell^™^ 96-Well Optical-bottom black chimney plates with polymer base (Cat. No. 152028) for all fluorescent stains. After staining with each fluorescent stain (below) on a separate plate, cells were imaged using a Tecan Infinite M200 fluorescence microplate reader. Due to differing growth rates of each cell line, cells were counted after each experiment using a Countess II FL cell counter and data was normalized to a total of 20,000 cells per well.

### Reduced glutathione

ThiolTracker^™^ Violet Kit (Glutathione Detection Reagent, Molecular Probes^®^ Cat. No. T10095) was used to measure concentration of reduced glutathione (GSH). GSH reduces the oxidized form of the enzyme GPx, which in turn reduces H_2_O_2_. Cells were rinsed with sterile PBS twice and 20 μM ThiolTracker was added to each well. Plates were incubated at 37 °C, 5% CO_2_/O_2_ for 30 min. Cells were, then, washed three times with sterile PBS, and imaged in phenol red-free FluoroBrite DMEM. Excitation and emission were measured in the violet spectrum at 404/526 nm, respectively [35].

### RS production

CellROX^®^ Oxidative Stress Reagents Kit (Molecular Probes^®^ Cat No. 10444) was used to measure RS production. CellROX reagent was added directly to the serum-free medium at a concentration of 5 μM to cells and incubated at 37 °C, 5% CO_2_/O_2_ for 30 min. Cells were, then, washed three times with sterile PBS, and imaged in phenol red-free FluoroBrite DMEM. Excitation and emission were in the green spectrum at 488/530 nm, respectively [36].

### Mitochondrial content

Mitochondrial content was measured using MitoTracker^®^ Mitochondrion-Selective Probes (Molecular Probes^®^ Cat No. M22426). Cells were stained with a 20 nM of MitoTracker Deep-Red and incubated at 37 °C, 5% CO_2_/O_2_ for 60 min. Cells were, then, washed three times with sterile PBS and imaged in phenol red-free FluoroBrite DMEM. MitoTracker^®^ excitation and emission were in the red spectrum at 635/670 nm [37].

### Lipid peroxidation damage

Lipid peroxidation (LPO) was measured with the Image-iT^®^ Lipid Peroxidation Kit based on the BODIPY^®^ 581⁄591 reagent (Cat. No. C10445). The ratio between red to green fluorescence indicates the degree of lipid peroxidation. Cells were stained with 10 μM of component A and incubated at 37 °C, 5% CO_2_/O_2_ for 30 min. Cells were, then, washed three times with sterile PBS and imaged in phenol red-free FluoroBrite DMEM. LPO red excitation and emission were 575/610 nm, respectively, and LPO green was 488/525, respectively [38].

### DNA isolation and 8-OHdG quantification

DNA from primary fibroblast cells was isolated using the Invitrogen DNAzol Reagent (Cat. No. 10503027). 100% Ethanol was then used to precipitate the DNA from the lysate, and finally solubilized in 8 mM NaOH, with its pH adjusted with 0.1 M HEPES [39]. After DNA was isolated from cells, each DNA sample was quantified using a Thermo Scientific NanoDrop 1000 Spectrophotometer.

To ensure all samples had the same concentration of DNA, DNA concentrations were standardized by dilution with dH2O. The Cell Biolabs, Inc. OxiSelectTM Oxidative DNA Damage ELISA Kit (8-OHdG Quantitation, Cat. No. STA-320-5) was used to measure the levels of DNA damage in DNA isolated from primary fibroblast cells [40]. We used a TECAN Infinite M200 plate reader set at 450 nm to analyze the ELISA plate, and a standard curve was generated to use in calculating the quantity of 8-OHdG in each well. Samples sizes for this measurement were as follows: small breed puppies N = 27, small breed old N = 16, large breed puppies N = 52, large breed old N = 25.

### Statistics

Data from every assay were first tested for normality using a Kolmogorov-Smirnov test. If not normal, the data were log-transformed, and re-tested using a Kolmogorov-Smirnov test prior to other statistical analyses to meet assumptions of an ANOVA. These data were then analyzed using a two-way ANOVA with body size (small or large, as stated above) and age (young or old) as the two factors. Results were considered significant if the corrected p-value was less than 0.05. To control for any false positives due to multiple comparisons, we have used Benjamini and Hochberg p-value adjustments [41] within all models, ANOVAs and generalized models. We performed the ANOVAs and corrected p-values in cran R [42]. Statistical models were also fit in cran R using the “bestglm” package [43]. The models were fit is using best-subset model selection according to the Bayesian information criterion (BIC) [44] via complete enumeration [45] for each model. When interactions were present, we calculated marginal effects using the “margins” package for cran R [46]. For all data including puppies, we present our results in two ways: (1) including all puppies (new-born to 3 months), and (2) including very young dogs (≤ 5 days old) to avoid any confounding differences potentially coming from IGF-1 secretion. In addition to our cellular parameters, we included data from [9] on mean breed lifespan in our statistical models. We considered “reasons for euthanasia” for all parameters involving older, euthanized dogs but it was never selected during the model building process due to the smaller sample size and variety of causes of euthanasia. Thus, despite varying reasons of euthanasia, our parameters do not seem to demonstrate an altering pathological phenotype or to have affected organismal physiology.

### Limitations

Due to the nature of working with pet dogs, our sample collection was limited in several ways. First, the number of puppies per breed or group was limited to those breeds that are altered as a breed requirement (tail docks and dewclaw removal). The number of older euthanized dogs was limited due to the owners’ willingness to provide us ear clips at the time of euthanasia. Secondly, we were not given complete medical charts for any dogs included in this study, thus, we have no records of diet, or exercise, which may be variants in OCR, ECAR and oxidative stress parameters. At times, veterinarians did not provide sex or weight for puppies, either. We do know that none of the dogs included in this study were taking any medications and none were obese. Additionally, primary fibroblast data can be noisy due to the heterogeneous nature of the isolated population of cells, though we corrected for heterogeneity of tissue of origin within our statistical models (See supplementary materials).

It should be noted that since this is an observational study and not a clinical trial, we could not establish causality but merely make observations about associations in the data. We hope to provide information about these associations that might lead to further inquiry that can corroborate the work here and possibly demonstrate causality.

## Results

### Whole-animal measurements

Our study included data from cells obtained from 21 breeds/mixes of large sized puppies and 11 breeds/mixes of small sized puppies (Table 1). Similarly, we gathered data from 22 breeds/mixes of large sized old dogs, 15 breeds/mixes of small sized old dogs (Table 2). Small breed old dogs averaged body masses of 7.04 ± 0.54 kg and ranged from 4.12 to 11.20 kg, whereas large breed old dogs averaged body masses of 31.04 ± 1.42 kg and ranged from 15.87 to 47.17 kg. Age at euthanasia was provided by veterinarians for all older dogs. Small breed dogs included in our study had significantly longer lifespans of 13.76 ± 0.38 years compared with large breed old dogs that lived 10.99 ± 0.47 years (ANOVA: F = 21.12, p < 0.001).

### Oxygen consumption rates (OCRs)

**Fig 1:**
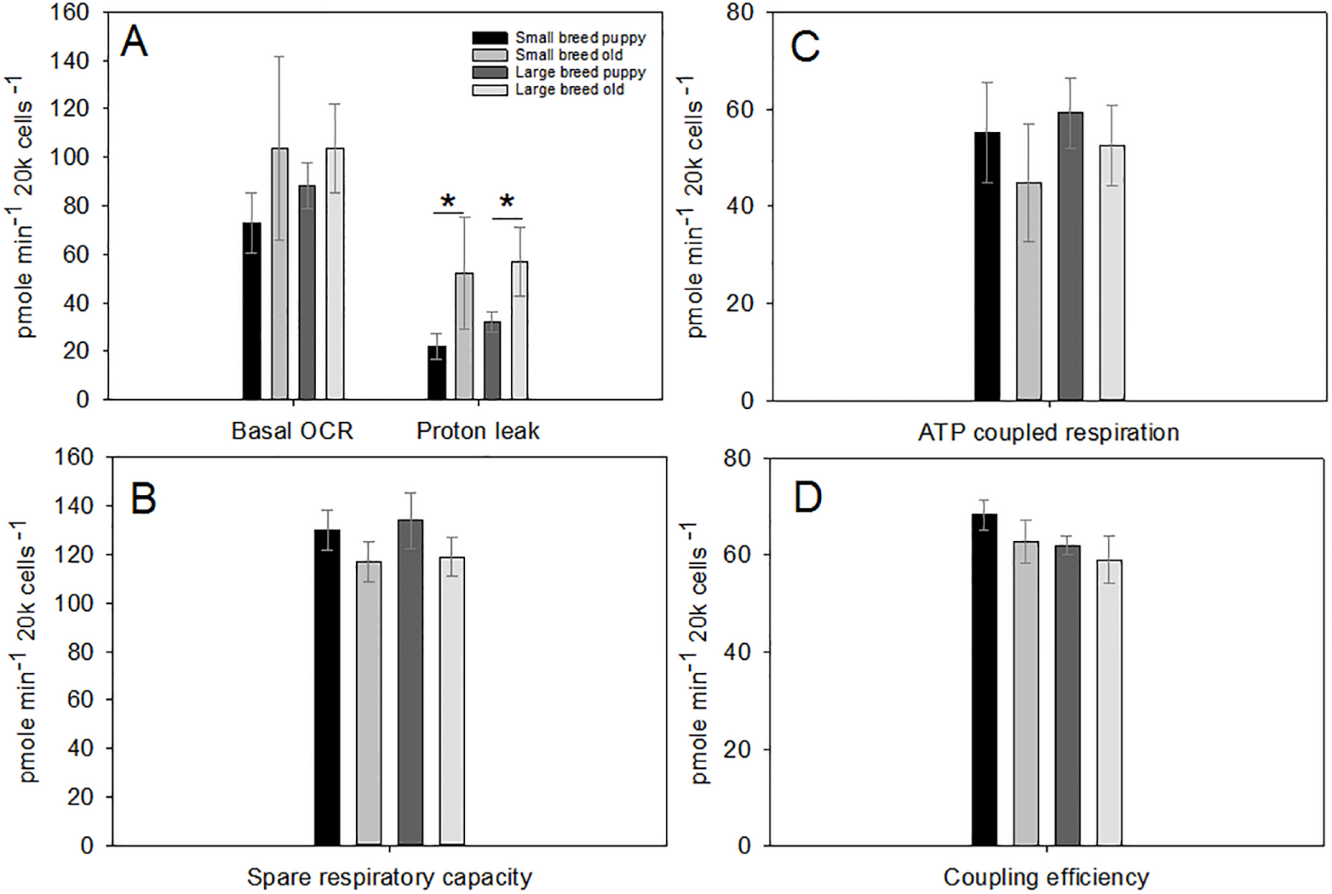
Panel (A) shows basal oxygen consumption rates (OCR) and measurements of proton leak after an injection with Oligomycin for each of our size and age groups. Panel (B) shows spare respiratory capacity as the difference between basal respiration and FCCP-induced maximal respiration. Panel (C) shows ATP coupled respiration as the difference between basal respiration, and oligomycin sensitive respiration. Panel (D) shows coupling efficiency calculated as the ratio of ATP production and basal respiration. For all parameters samples sizes can be found in Tables 1 and 2. Values are presented as averages ± SEM. Asterisk (*) highlight significant differences. Proton leak shows size class differences.

Using a two-way ANOVA, we found no significant differences between small and large size classes in most OCR parameters. Though, there is a marginal difference in basal OCR (Table 3; Fig 1), and a significant difference in proton leak. We found no significant differences between puppy and old age classes in any OCR parameters.

**Table 3:** Two-way ANOVA results for every parameter measured.

Measured Parameter	ANOVA factor	Corrected p-value
Basal OCR	Age class	0.371
	Size class	0.063
	age x size	0.913
Proton leak	Age class	0.126
	Size class	0.013*
	age x size	0.942
ATP coupled respiration	Age class	0.528
	Size class	0.525
	age x size	0.705
Spare respiratory capacity	Age class	0.913
	Size class	0.913
	age x size	0.913
Coupling efficiency	Age class	0.559
	Size class	0.209
	age x size	0.743
Total Glycolysis	Age class	0.062
	Size class	0.010*
	age x size	0.728
Total glycolytic capacity	Age class	0.19
	Size class	0.002*
	age x size	0.55
Non-glycolytic acidification	Age class	0.917
	Size class	0.001*
	age x size	0.917
GSH	Age class	<0.001*
	Size class	0.419
	age x size	0.948
RS production	Age class	0.714
	Size class	0.714
	age x size	0.714
Mitochondrial content	Age class	0.300
	Size class	0.619
	age x size	0.855
LPO damage	Age class	0.306
	Size class	0.919
	age x size	0.919
8-Ohdg content	Age class	0.264
	Size class	0.429
	age x size	0.39

Asterisks (*) highlight significant differences.

Within our statistical models, when very young puppies are considered, there is a marginal positive relationship between weight and basal OCR, and a significantly negative relationship between basal OCR and mean breed lifespan and age (Table W in S1 File). Older dogs showed a significantly negative correlation with basal OCR and age (Table X in S1 File). Proton leak was negatively correlated with breed lifespan in very young puppies (Table Z in S1 File), and positively correlated with weight in older dogs (Table Aa in S1 File). When only very young puppies are considered, there is a significant negative correlation between age and ATP coupled respiration (Table Ac in S1 File). In older dogs, there is a significantly negative relationship between age and ATP coupled respiration (Table Ad in S1 File). When all puppies are considered, we see a significantly positive relationship between mean breed lifespan and spare respiratory capacity (Tables Ae, Af in S1 File). All puppies showed a significantly negative relationship between coupling efficiency and age and mean breed lifespan (Table Ah in S1 File). When only very young puppies are considered, there is a significantly negative relationship between coupling efficiency and age and a positive relationship between coupling efficiency and breed lifespan (Table Ai in S1 File).

### Extracellular acidification rate (ECAR)

**Fig 2:**
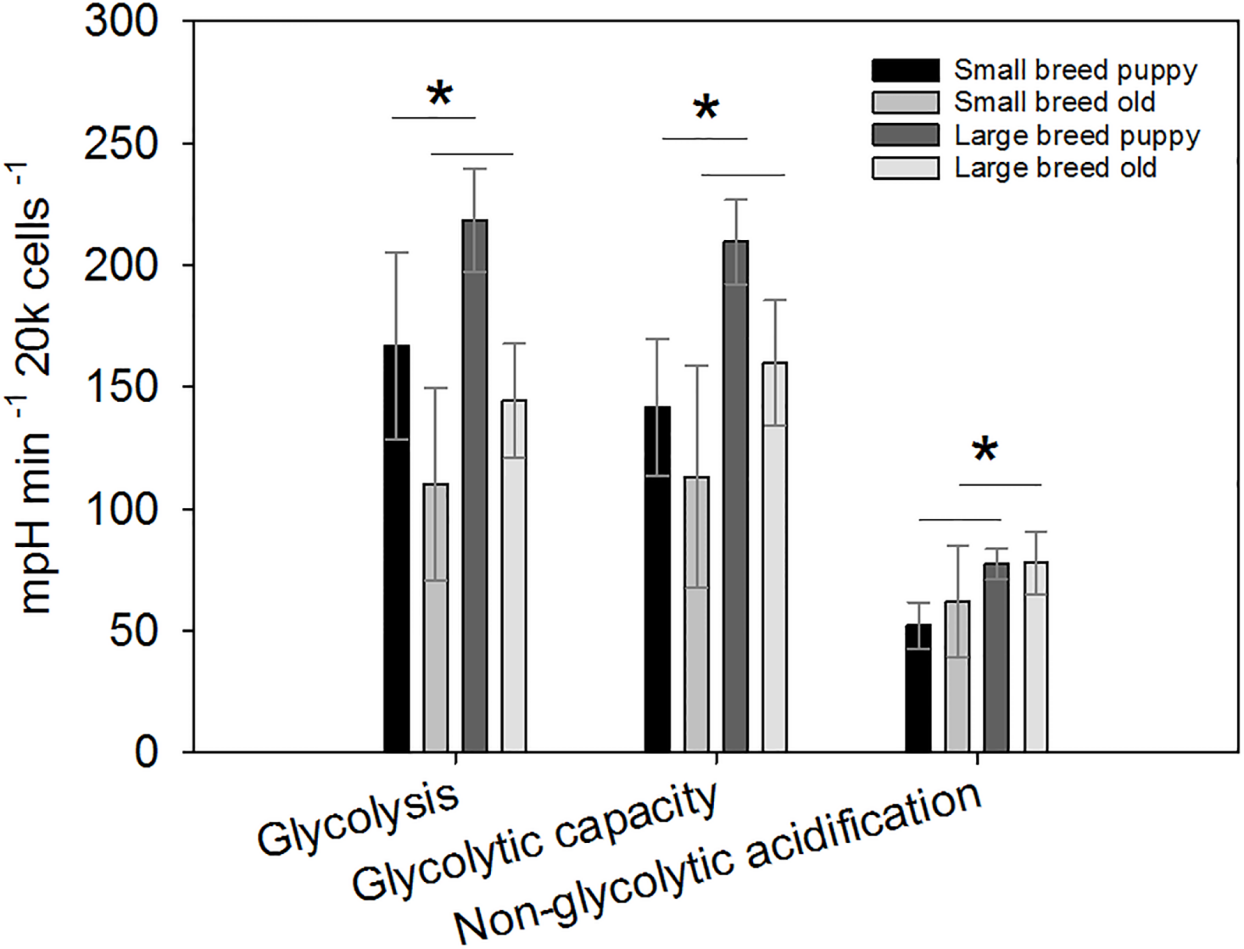
Panel (A) shows glycolysis rates after the injection with glucose. Panel (B) shows potential glycolytic capacity after the injection with Oligomycin, and Panel (C) shows non-glycolytic acidification after the injection of 2-DG. For all parameters samples sizes can be found in Tables 1 and 2. Values are presented as averages ± SEM. Asterisk (*) highlight significant differences. Every glycolytic parameter shows size class differences.

Using a two-way ANOVA, we found significant differences after p-value correction between small and large size classes in glycolysis and, glycolytic capacity where large breed puppies had significantly higher rates compared to small breed puppies and older dogs, and non-glycolytic acidification, where each of these parameters were higher in large breed dogs (Table 3; Fig 2).

We found no significant differences between puppy and old age classes in any glycolytic parameter (Table 3).

Within our statistical models, in all puppies and very young puppies, we see a significantly higher total glycolytic capacity in large breed dogs compared with small breeds (Tables An, Ao in S1 File). In older dogs, we see a significantly negative relationship between breed lifespan and total glycolytic capacity (Table Ap in S1 File). When all puppies are considered, large breed dogs show a significantly higher non-glycolytic acidification (Table Aq in S1 File). When only very young puppies are considered, large breed dogs still show a significantly higher non-glycolytic acidification and there is a significantly negative correlation between non-glycolytic acidification and mean breed lifespan (Table Ar in S1 File). In older dogs, there is a significantly negative correlation between mean breed lifespan and non-glycolytic acidification (Table As in S1 File).

### Oxidative stress profiles

**Fig 3:**
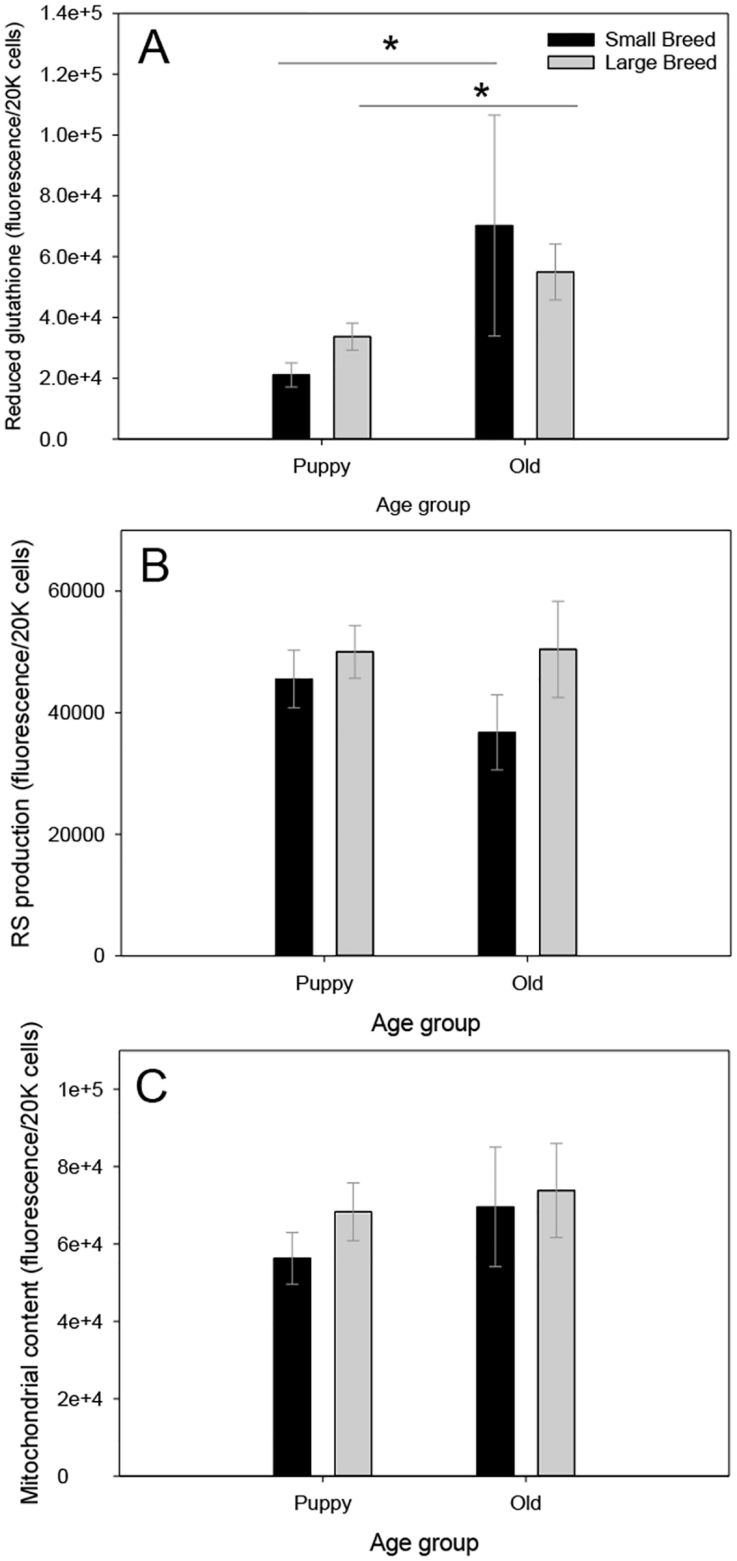
Panel (A) shows concentration of reduced glutathione (GSH). Panel (B) shows basal RS production. Panel (C) shows mitochondrial content in primary fibroblasts. For all parameters samples sizes can be found in Tables 1 and 2. Values are presented as averages ± SEM. Asterisk (*) highlight significant differences. GSH shows an age class difference.

**Fig 4:**
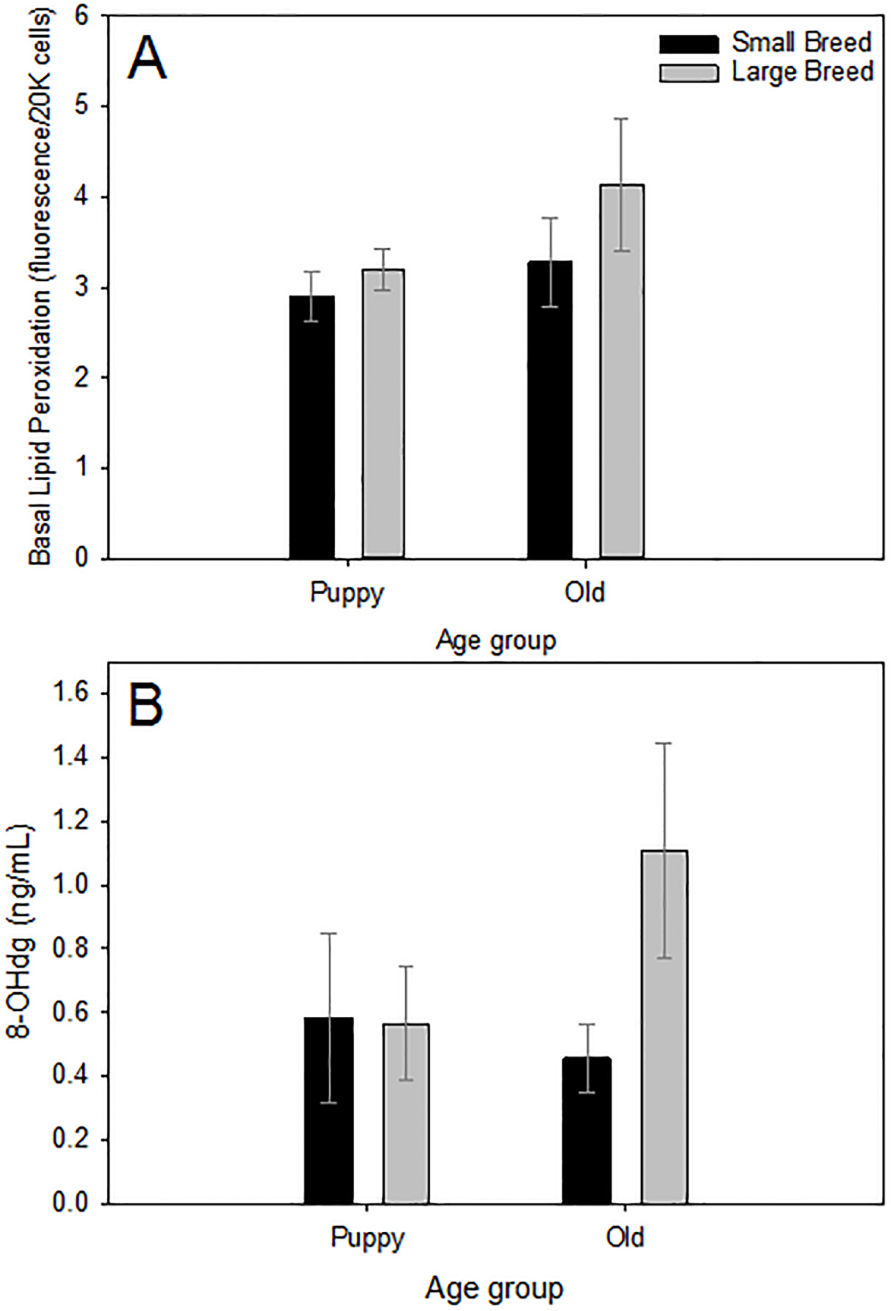
Panel (A) shows lipid peroxidation damage and panel (B) shows oxidative DNA damage measured by quantifying 8-OHdg. For all parameters included in LPO damage, samples sizes can be found in Tables 1 and 2. Samples sizes for DNA damage are as follows: small breed puppies N = 27, small breed old N = 16, large breed puppies N = 52, large breed old N = 25. Values are presented as averages ± SEM. Asterisk (*) highlight significant differences.

Using a two-way ANOVA, we found no significant differences between small and large size classes in oxidative stress parameters (Table 3; Figs 3 and 4). We found significant differences between puppy and old age classes in GSH (p < 0.001; Fig 3A) where older dogs had significantly higher concentrations of GSH compared with puppies, but no age class differences in other oxidative stress parameters were found (Table 3; Figs 3 and 4).

Within our statistical models, for very young puppies there are statistically significant positive correlations between GSH and age as well as weight (Table G in S1 File). All female puppies and very young female puppies showed a significantly higher RS production (Tables I, J in S1 File), and there was a marginally significant negative correlation between mean breed lifespan and RS production for older dogs (Table J in S1 File). Mitochondrial content of very young puppies showed a positive relationship with age (Table M in S1 File). We found a marginally negatively correlation between DNA damage and mean breed lifespan (Tables R, S in S1 File).

## Discussion

We found that basal respiration (marginally) and proton leak were significantly higher in older dogs compared with puppies (Fig 1). Thus, our data suggests that cellular oxygen consumption changes with age similarly in both size classes. We also found differences between small and large size classes with respect to glycolytic parameters, where larger breed puppies have significantly higher glycolytic capacity compared with smaller breeds (Fig 2). Similarly, non-glycolytic acidification increased significantly in older dogs despite of their size class. In other studies, lactate dehydrogenase, the enzyme that catalyses the conversion of anaerobically produced lactate to pyruvic acid, increases with age in blood from aging Beagles [47], and urine lactate also increases with age in Labradors [48], an indication that increases in glycolytic pathways with age have been previously described in the literature, however, neither of the above studies compared the concentration of this enzyme across breed sizes Taken together, our metabolic rate data may suggest that oxygen consumption increases with aging in dogs, while glycolysis decreases. Though, our ATP coupled respiration data suggests that efficiency in oxidative pathways may slow, without a concomitant increase in glycolysis, potentially decreasing cellular productivity. ATP levels are often associated with apoptotic or necrotic pathways, so that, if ATP levels drop significantly necrosis may occur. Many cell types that are or become cancerous, are often associated with a more glycolytic phenotype for ATP [49]. A conundrum with large breed dogs and cancer is that large dogs also secrete more IGF-I relative to smaller breeds, which accounts for their rapid growth and large size [50]. Unfortunately, IGF-I also promotes tumorigenesis [51]. Interestingly, large breed puppies tended to have an increase in glycolysis (Fig 2). This is further cofounded by the finding that, in older dogs, shorter-lived breeds have significantly higher glycolytic phenotypes, thus, from puppies to seniors, large breed/shorter-lived dogs seems to have a persistent glycolytic phenotype. This may suggest that cells from the larger breeds may be metabolically pre-disposed to taking on a tumor-like phenotype. In a study looking at dog morbidities in across different breeds and size classes, small breed dogs showed no significant effect of age on morbidities compared with large breed dogs. The authors suggest that there may be a physiological basis that allows small breed dogs to reduce the effect of age on multiple morbidities [52], our data may provide a physiological mechanism to underscores the whole-organism pattern described by [52]. A study comparing mice with accelerated and delayed aging found a suppression of the IGF-1/GH axis and metabolism (including glycolysis) to be associated with delayed aging [53], a pattern that may be similar in small breed dogs.

Oxidative stress encompasses not only the rate of RS production, but also the antioxidant system and the oxidative damage accumulated when the antioxidant system cannot keep up with RS production [54]. There is evidence that antioxidant enzymes are under endocrine control and can be affected by sex and age of the dog [55]. Thus, as predicted, we found a significant age class difference in GSH, where older dogs, despite their size class, showed an increase in GSH. Though, others have found that in older beagles, especially males, there was a decrease in GSH [56], these conflicting results to ours, however, were measured in blood and in a single breed. Another study also found a significant decrease in GSH in older dogs compared with younger ones; however, this difference was confounded by sex differences, where there was a steeper decline in the activity of this enzyme in male dogs compared with female dogs. Additionally, this study included a narrow range in body sizes of dogs [57]. In contrast, we found a significant increase in GSH in older dogs compared with younger ones in a broader range of breeds and sizes. Similar to our findings, in the dog brain, there are age-related increases in oxidative damage to lipids and proteins, although GSH also increased [58]. However, we also see a significant increase in RS production in female puppies, thus, a GSH increase later in life could be a way to help offset damage from increases in RS production early-on. Other studies have also found sex-differences in the level of enzymatic antioxidant activity in the blood exist so that older females have higher reduced glutathione and SOD activity [56, 59]. Though, we do not see a significant reduction in GSH in large breed dogs, there may be other antioxidant enzymes that are being reduced in function and, thus, allowing for an accumulation of oxidative damage in larger breed dogs. Others have shown an age-related increase in SOD activity, another antioxidant enzyme measured in blood [55]. Surprisingly, we found no differences in average RS production across size and age classes in primary fibroblasts of dogs, though it is common to assume that with age, RS production should increase [60], many studies have not shown a strong correlation between RS production and lifespan [61]. However, we saw that longer-lived breeds may have a reduction in RS production when they are puppies. Artificial selection for longer growth trajectories in larger dogs could lead to developmental diseases that decrease lifespan [27]. As stated in [9], some authors have suggested that longer developmental trajectories are associated with increases in RS production. Larger breeds could be burdened with increases in oxidative damage for prolonged periods of time during early life, leading to higher rates of diseases associated with free radical damage, and hence early mortality. If we couple our findings of increases in RS production in shorter-lived breeds coupled with and a more glycolytic phenotype in larger breeds, these two pathways provide a mechanism for some of these developmental diseases in shorter-lived breeds compared with longer-lived breeds. In mammals, a shift from growth to somatic maintenance (such as that done earlier by small breed dogs), may function as a tumor-suppression mechanism [62]. It is noteworthy to mention that RS are not produced at a constant rate and that the production of RS can alter with changing physiological state, such as mitochondrial redox state and membrane potential, intra-mitochondrial RS scavenging, substrate input, etc. [63]. There are a number of extrinsic factors, such as exercise, and diet, and intrinsic factors such as growth rate, which can affect levels of RS production and, thus, oxidative stress in dogs. Some authors have found that RS production differs in different organs [24], thus, RS production in primary fibroblast cells may not be representative of the whole animal at all points in time, however, aging studies in mice have found that in the liver, spleen, lungs, and kidney most genes associated with aging showed similar expression, indicating a systemic response to aging [53], which alludes to primary fibroblast being potentially representative of the whole-animal phenotype. Additionally, other studies have found oxidative stress links to aging in different sized dogs. The prevalence of age-related cataract formation (ARC), an aging defect partly linked to oxidative stress, was significantly lower in small dog breeds compared with giant breeds [64].

We found a marginally significantly negative correlation between DNA damage measured by quantifying 8-OHdg concentration and mean breed lifespan. We found that the median for this data set in the “old” age class were different demonstrating an empirical difference; however, there is no statistical test to test median interaction. We had a smaller sample size for this parameter, thus, we propose that a large samples size would correct for outliers. Oxidative damage to DNA can decrease the lifespan of cells and tissues through the loss or modification of genes [61]. It seems that the literature is congruent on the fact that long-lived animals are better able to repair DNA compared with short-lived ones [61, 65]. DNA repair systems are extensive and energetically expensive to maintain in terms of ATP production, thus, longer-lived species tend to spend more energy maintaining this system [65]. Because oxidative DNA damage can be so detrimental, organisms have evolved a complicated system for DNA repair [22]. RS-mediated DNA damage is usually a double-strand lesion, which can lead to genome rearrangements, a process of which cancer is often associated [22]. To deal with this kind of damage, cells have base excision repair, transcription-coupled repair, and nucleotide excision repair mechanisms [22], all dictated by enzymes, such as endonucleases and exonucleases [21, 64]. Repair potential in adult animals seems to be determined during embryogenesis and ultimately correlated with the organism’s lifespan [21]. 8-OHdg is one of the main DNA modifications produced by RS and has shown a positive correlation with age in metabolically active tissues of rats [66, 67]. If we assume that the accumulation of 8-OHdg is a life-long, steady process, and that the rate of accumulation represents a balance between accumulation and repair, then, considering our data, we suggest that DNA repair mechanisms seem to be failing earlier in larger breed dogs, thus, more DNA damage is accumulated in these breeds sooner. Since we found no differences in RS production with age class or size class, this also suggests that the repair mechanisms, the endonucleases and exonucleases responsible for fixing DNA oxidative damage are decreasing their activity with age.

Aging is an inherently complex phenomenon which leads to a whole-animal phenotype change with increasing age, involving multiple mechanisms at several levels of organization [3]. Thus, while some aspects of oxidative stress may be driving longevity differences in small and large breed of dogs, there may be other mechanisms dictating these differences in lifespan. Although, cellular work in dogs is limited, primary dermal fibroblasts growth potential was inversely associated with mortality, with the exception of Chihuahuas, Great Danes and Irish Wolfhounds [68]. However, growth rate of primary dermal fibroblasts in cell culture may be inversely proportional to the age of the animal they were isolated from [69] and Li and co-authors did not control for the age of the dog from which they isolated primary fibroblast cells, thus potentially confounding the results of this study. Another component of cellular physiology that may be linked to the rate of aging is telomere length and rate of shortening. Telomeres maintain genomic integrity by protecting chromosome ends and tend to shorten with each cell division [70]. Since cellular division increases with age, we would expect a decrease in telomere length as animals increase in age, but at possibly different rates according to body mass. We know that telomere lengths decrease with age for certain breeds of dogs [71, 72], but not others [70]. The retriever group, a larger bodied breed, seems to trend towards a decrease in telomere length with increasing age [70]. Whereas, oxidative stress and telomere length can be linked, other aging theories such as the altered protein theory, which accounts of the accumulation of damaged proteins within cells or the network theories of aging [3], and epigenetics can also have effects on the rates of aging between small and large breed dogs. However, within our study, we found that all aspects of glycolysis were significantly higher in larger breeds compared with smaller breeds, GSH was higher in older dogs, despite of size class, and that RS production showed a negative correlation and DNA damage seem to be positively correlated to breed lifespan. Thus, our results provide evidence for potential mechanisms that could be involved in the differences in lifespans between small and large breeds of dogs.

## Supporting information

S1 File Results from statistical analysis for every parameter measured. (PDF)
