# Supplementary material for: Cellular metabolism and oxidative stress as a possible determinant for longevity in small breed and large breed dogs
Source: PLoS One. 2018 Apr 25;13(4):e0195832. doi: 10.1371/journal.pone.0195832 (PMC5918822; doi:10.1371/journal.pone.0195832)
Supplement: S1 File — (PDF) [file pone.0195832.s001.pdf]

Supplement: Cellular metabolism and oxidative stress as a possible determinant for longevity in small breed and large breed dogs

Ana Gabriela Jimenez
Colgate University
Department of Biology

Josh Winward
Colgate University
Department of Biology

Ursula Beattie
Colgate University Department of Biology

William Cipolli III*
Colgate University
Department of Mathematics
will@cipolli.com

1 Description of Data

These data consist of a variety of measurements on 282 dogs. Only one observation was sourced from an amputation and thus was removed for data analysis. Of the remaining 281 dogs there are 50 old dogs (aged 6 to 16 years) and 231 puppies (aged newborn to 36 months), 101 females and 120 males, and representation of 65 breeds. For each dog, we attempted to capture the following data:

- breed
- sex
- spayed/neutered status
- age
- reason for euthanasia (when applicable)
- weight
- reduced glutathione (GSH) (flu/20K cells)
- reactive species (RS) (flu/20k cells)
- mitochondrial content (MC) (flu/20k cells)
- lipid peroxidation damage (LPO) (flu/ 20k cells)
- DNA damage (ng/mL)
- Basal OCR (per 20K cells)
- proton leak (per 20K cells)
- ATP production (ATP) (per 20K cells)
- spare respiratory capacity (per 20K cells)
- coupling efficiency (per 20K cells)
- total Glycolysis (per 20K cells)
- total Glycolytic capacity (per 20K cells)
- Non-Glycolitic acidification (per 20K cells).

Due to the nature of these data, which were compiled from many sources, and the cost of certain measurements, we do not have complete cases for all dogs in the sample. For each model, we note how many such observations were left out during model building and fitting procedures in the tables with those resulting models. Information about such missingness is detailed below.

*All R Code and data can be provided by request to will@cipolli.com

- 31 dogs are missing spayed/neutered
- 60 dogs are missing sex
- 231 dogs are missing reason for euthanasia
- 23 dogs are missing weight
- 162 dogs are missing the DNA damage (ng/mL)

  Remark: this is an expensive measurement
- 1 dog is missing the coupling efficiency (/20K cells)

A summary of the categorical variables of the 231 puppies,

- 76 female, 95 male, 60 unknown
- 0 were fixed, 209 were not fixed, 22 unknown
- 162 were large breeds, 69 were small breeds
- 65 were dewclaw, 10 were ear clips, 156 were tail docks

and of the 50 old dogs,

- 25 female, 25 male
- 39 were fixed, 2 were not fixed, 9 unknown
- 32 were large breeds, 18 were small breeds
- all observations were from ear clips.

The source of the sample and the spay/neutered status of a dog are confounded with the age classification – dogs that were not fixed are almost exclusively puppies and only puppies had samples from dewclaws or tail docks. So that we might still consider the effect of these variables we complete a stratified analysis creating two strata on age class. Additionally, we explore the difference between newborn puppies and those puppies older than one month.

A summary of responses for puppies is provided in Table A and a summary of responses for adult dogs is in Table B. We see, as indicated by the difference in the mean and median, and Figure 1 and Figure 2, that many of the variables of interest are heavily skewed.

Table A: Summary of response data for puppies.

Measurement	Obs.	Median	IQR	Mean	Standard Deviation
GSH (flu/20K cells)	231	10878.11	23593.21	29962.39	50700.56
RS (flu/20k cells)	231	32875.16	36296.37	48823.85	51126.31
MC (flu/20k cells)	50	51354.82	76161.27	72291.94	66940.82
LPO (flu/ 20k cells)	231	2.09	3.38	3.12	2.76
DNA damage (ng/mL)	78	0.14	0.35	0.58	1.31
Basal OCR (per 20K cells)	231	43.98	89.28	83.81	114.79
Proton leak (per 20K cells)	231	10.99	23.7	29.18	48.57
ATP (per 20K cells)	231	22.33	68.06	58.32	90.67
Spare respiratory capacity (per 20K cells)	231	110.4	66.67	133.01	129.63
Coupling efficiency (per 20K cells)	230	66.03	39.62	63.92	25.4
Total Glycolysis (per 20K cells)	231	117.16	218.06	203.57	286.74
Total Glycolytic capacity (per 20K cells)	231	113.16	232.78	189.84	226.87
Non-Glycolitic acidification (per 20K cells)	231	46.33	77.88	69.93	80.83

Table B: Summary of response data for old dogs.

Measurement	Obs.	Median	IQR	Mean	Standard Deviation
GSH (flu/20K cells)	50	25962.06	60167.39	60474.16	100070.9
RS (flu/20k cells)	50	29634.28	43696.09	45489.44	39444.08
MC (flu/20k cells)	50	51354.82	76161.27	72291.94	66940.82
LPO (flu/ 20k cells)	50	2.46	3	3.82	3.56
DNA damage (ng/mL)	41	0.43	0.67	0.85	1.38
Basal OCR (per 20K cells)	50	70.7	100.54	103.64	126.24
Proton leak (per 20K cells)	50	17.43	48.48	55.18	86.05
ATP (per 20K cells)	50	34.06	65.15	49.78	47.9
Spare respiratory capacity (per 20K cells)	50	114.24	61.38	118.33	42.5
Coupling efficiency (per 20K cells)	50	58.55	33.31	60.4	25.06
Total Glycolysis (per 20K cells)	50	74.24	163.72	132.01	145.69
Total Glycolytic capacity (per 20K cells)	50	80.82	190.63	143.04	163.88
Non-Glycolitic acidification (per 20K cells)	50	47.06	57.79	72.2	81.54

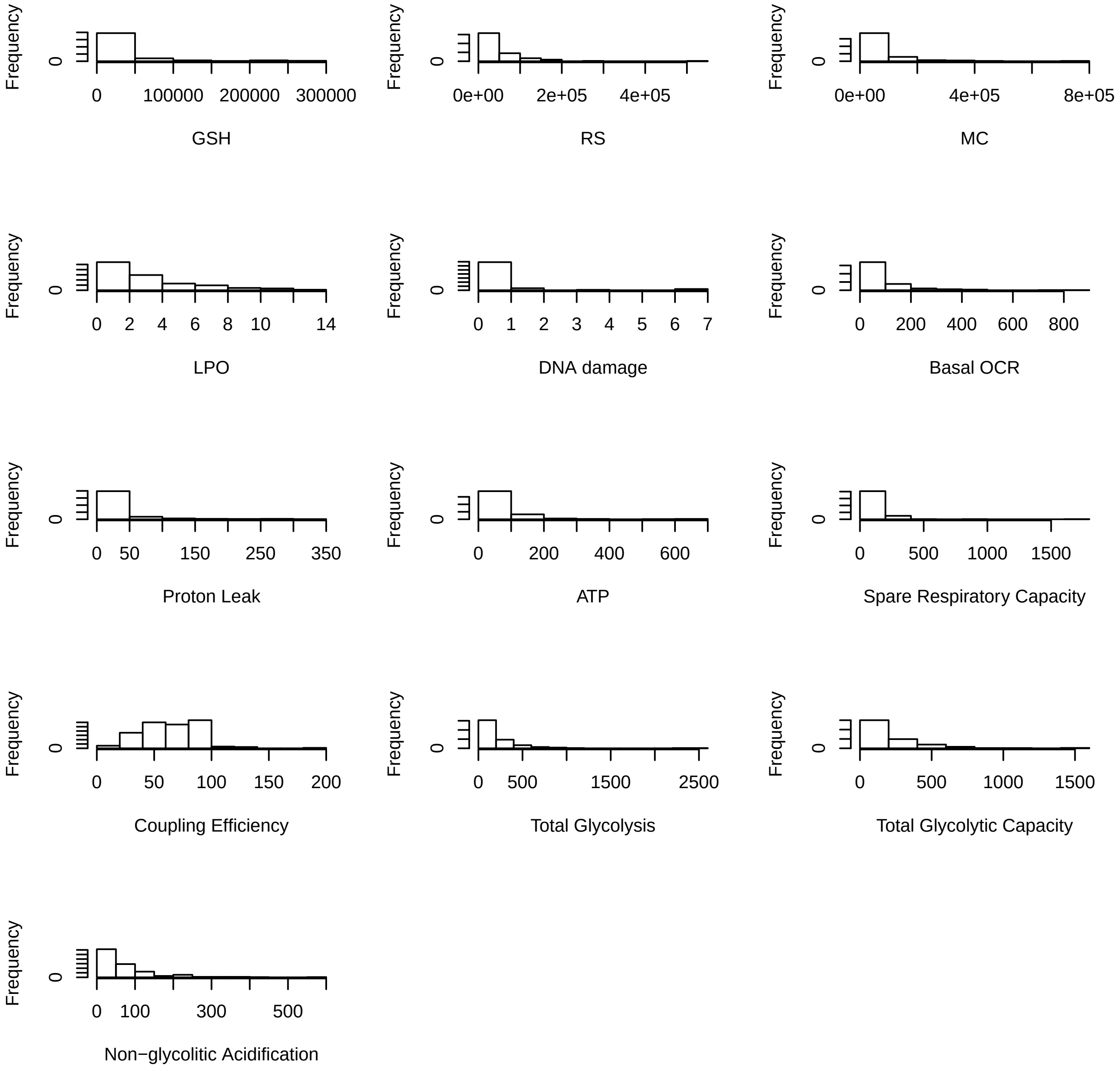

Figure 1: Histograms of response variables for puppies in the sample.

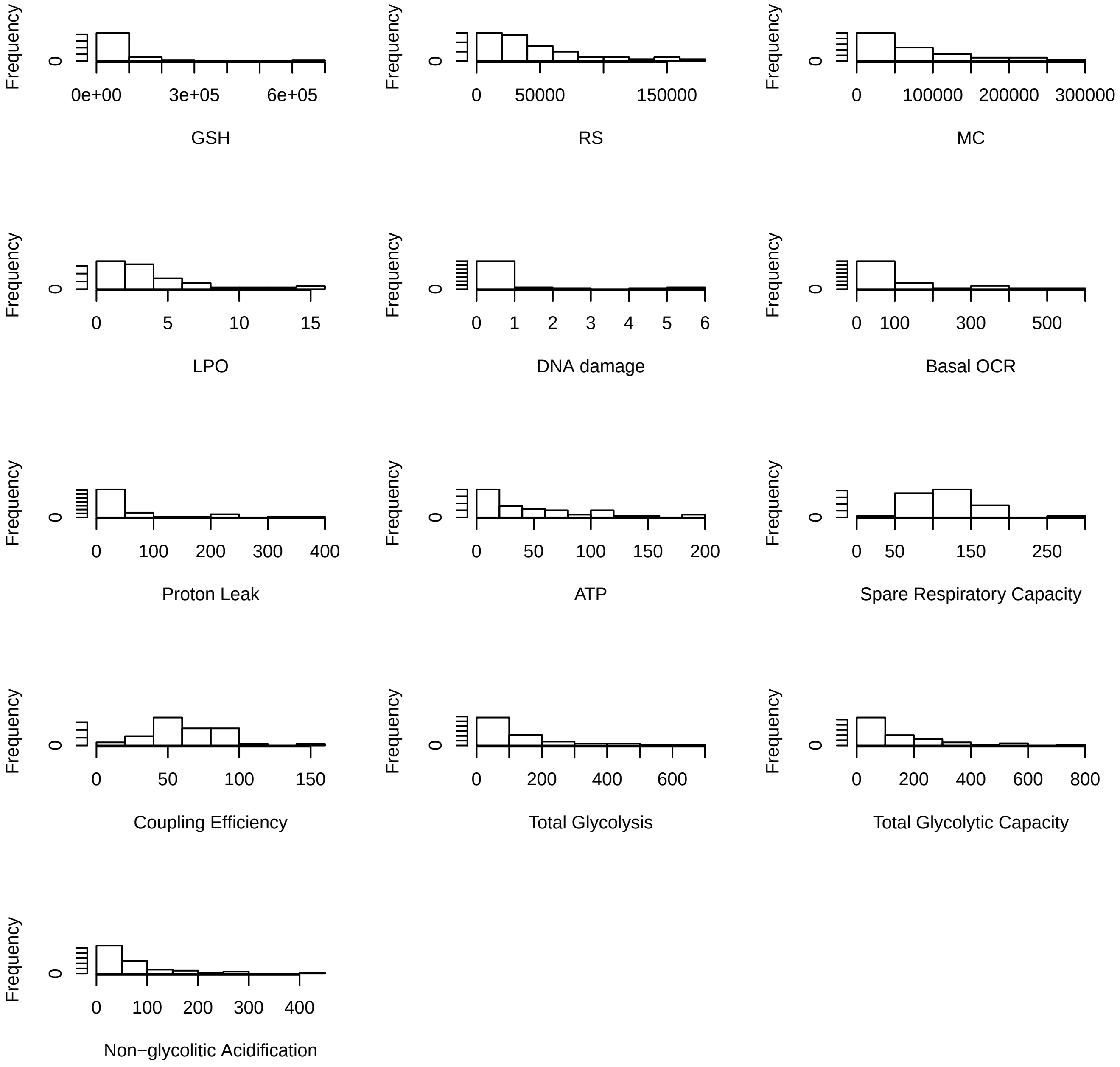

Figure 2: Histograms of response variables for puppies in the sample.

2 ANOVA Analysis

We completed a multivariate analysis of variance (MANOVA) which is a multivariate extension of a regular analysis of variance (ANOVA). The DNA damage variable is left out of this MANOVA due to the large amount of missing data.

Table C: Overall MANOVA results.

	Pillai	approx F	Pr(>F)
age class	0.132	3.346	<0.001
size	0.090	2.184	0.013
age class×size	0.013	0.286	0.991

The MANOVA results in Table C show significance for the multivariate analysis in age class and size so we probe each dependent variable separately using ANOVA below. We adjust the p-values within each model using the approach in Benjamini and Hochberg (1995) instead of the overly conservative Bonferonni adjustment (Dunn, 1961). This approach allows for the control the expected proportion of false discoveries instead of the probability of making at least one false discovery, thus preserving power.

The ANOVA results in Table D suggest significant differences in GSH across age classes, proton leak across size classes, total Glycolysis across size classes, and non-Glycolitic acidification across size classes. Additionally, we see marginal differences in Basal OCR across size, and total Glycolysis across age classes.

Table D: ANOVA for each variable.

		Sum Sq	Mean Sq	F value	Pr(>F)	Corrected
log(GSH)	age class	34.4	34.44	19.864	<0.001	<0.001
(n=281)	size	2.0	2.04	1.177	0.279	0.419
	age class×size	0.0	0.01	0.004	0.948	0.948
log(RS)	age class	0.11	0.114	0.135	0.714	0.714
(n=281)	size	0.17	0.174	0.206	0.650	0.714
	age class×size	0.37	0.367	0.435	0.510	0.714
log(MC)	age class	3.5	3.493	2.724	0.100	0.300
(n=281)	size	0.9	0.863	0.673	0.413	0.619
	age class×size	0.0	0.043	0.033	0.855	0.855
log(LPO)	age class	2.45	2.453	2.690	0.102	0.306
(n=281)	size	0.10	0.103	0.113	0.737	0.919
	age class×size	0.01	0.009	0.010	0.919	0.919
log(DNA Damage)	age class	0.625	0.625	2.959	0.088	0.264
(n=119)	size	0.133	0.133	0.631	0.429	0.429
	age class×size	0.270	0.271	1.282	0.260	0.390
log(Basal OCR)	age class	2.2	2.154	1.343	0.248	0.371
(n=281)	size	8.7	8.656	5.397	0.021	0.063
	age class×size	0.0	0.019	0.012	0.913	0.913
log(Proton leak)	age class	5.2	5.174	3.010	0.084	0.126
(n=281)	size	14.3	14.259	8.295	0.004	0.013
	age class×size	0.0	0.009	0.005	0.942	0.942
log(ATP)	age class	1.8	1.788	0.871	0.352	0.528
(n=281)	size	3.8	3.791	1.846	0.175	0.525
	age class×size	0.3	0.294	0.143	0.705	0.705
log(Spare respiratory capacity)	age class	0.00	0.003	0.012	0.913	0.913
(n=281)	size	509	509.2	0.797	0.373	0.913
	age class×size	0.01	0.013	0.050	0.822	0.913
Coupling Efficiency	age class	509.21	509.21	0.797	0.373	0.559
(n=280)	size	2122	2121.8	3.321	0.070	0.209
	age class×size	69	68.7	0.108	0.743	0.743
log(Total Glycolysis)	age class	6.1	6.144	4.206	0.041	0.062
(n=281)	size	12.9	12.900	8.831	0.003	0.010
	age class×size	0.2	0.177	0.121	0.728	0.728
log(Total Glycolytic capacity)	age class	4.0	4.048	2.346	0.127	0.190
(n=281)	size	20.2	20.214	11.714	<0.001	0.002
	age class×size	0.6	0.618	0.358	0.550	0.550
log(Non-Glycolitic acidification)	age class	0.0	0.018	0.011	0.917	0.917
(n=281)	size	21.4	21.411	12.702	<0.001	0.001
	age class×size	0.1	0.053	0.032	0.859	0.917

Note: we added one to the DNA damage measurements to avoid issues when performing the log transform of the response variable with measurements of zero.

3 Models

The models are fit in `cran R` (R Core Team, 2016) using the “bestglm” package (McLeod and Xu, 2017) to perform best-subset model selection according to the Bayesian information criterion (BIC) (Schwarz et al., 1978) via complete enumeration (Morgan and Tatar, 1972) for each model. BIC penalizes false positives more than false negatives when there are eight or more observations. Additionally, we adjust the p-values within each model using the approach of Benjamini and Hochberg (1995) as we did in the ANOVA analysis.

It should be noted that since this is an observational study and not a clinical trial we cannot establish causality but merely make observations about associations in the data. We hope to provide information about these associations that might lead to further inquiry that can corroborate the work here and possibly demonstrate causality.

Below, we discuss the models for GSH (flu/20K cells) in detail and provide the information necessary for such interpretations for the other response variables of interest.

3.1 Reduced Glutathione (GSH)

A log transform of GSH (flu/20K cells) was completed to correct issues with the diagnostic plots in the full model. Figure 3 shows the diagnostic plots for the pre-log-transform model and Figure 4 shows the diagnostic plots for the transformed model. Note that the “Residuals vs Fitted’ plot is more closely fit by a horizontal line at zero, without the megaphone pattern in the original, and the ‘Normal Q-Q’ plot fits well except for the tails.

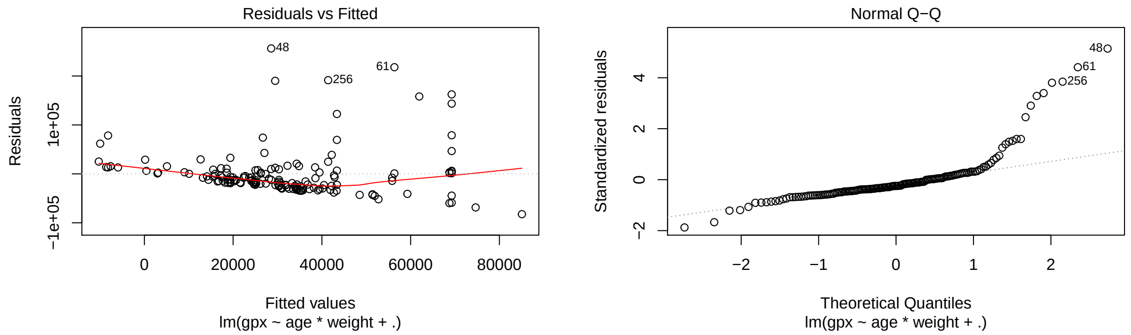

Figure 3: Diagnostic plots before the log-transform of GSH for puppies ($n = 159$).

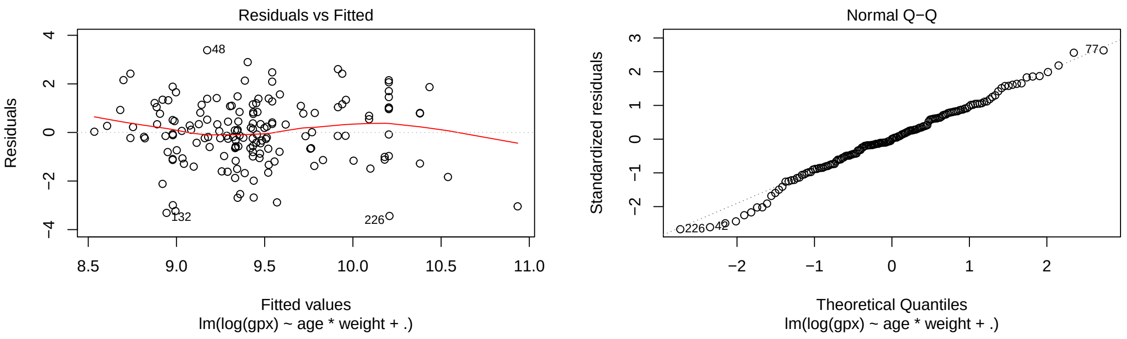

Figure 4: Diagnostic plots after the log-transform of GSH for puppies ($n = 159$).

After completing model building from the full set of explanatory variables, we are left with the following reduced model where none of the variables are considered significant.

Table E: The reduced model for GSH for Puppies ($n = 159$).

	Estimate	Std. Error	t value	$\Pr(>\|t\|)$
(Intercept)	9.443	0.110	86.12	<0.001

This yields a regression equation of,

$$log(\text{GSH}_i) = 9.4432 + \epsilon_i.$$

To be interpretable, in terms of the response variable, we solve this for GSH_i.

$$\text{GSH}_i = e^{9.4432} e^{\epsilon_i} = 12622.04\ e^{\epsilon_i}$$

In Figure 5, we can see that we have two clusters – puppies that are at most a week old and puppies that are 1.5 months or older. While these observations are representative of puppies, the group of slightly older dogs are influential to the modeling of certain responses.

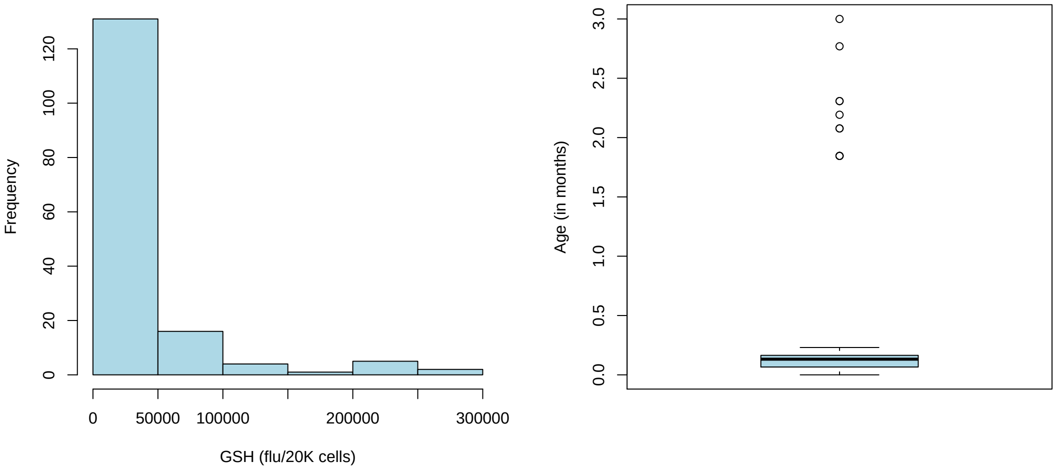

Figure 5: Histogram of GSH in puppies (left) and a box plot of age in puppies(right) ($n = 159$).

For example, consider the model fit to just the puppies less than a week old. The the interaction effect of age and weight is now a highly significant explanatory variable.

Table F: The reduced model for GSH in very young puppies ($n = 149$).

	Estimate	Std. Error	t value	$\Pr(> \lvert t \rvert)$	Corrected
(Intercept)	8.781	0.172	51.051	< 0.001	< 0.001
age×weight	15.211	3.178	4.786	< 0.001	< 0.001

This yields a regression equation of,

$$log(\text{GSH}_i) = 8.781 + 15.211 \text{ age} \times \text{weight} + \epsilon_i.$$

Interpretation of the interaction term is difficult as any change in age or weight are combined into one effect in the regression model. To make interpretations for age and weight we calculate the average marginal effects (AME) using the `margins` package (Leeper, 2017) in `cran R`.

Table G: The average marginal effects (AME) for GSH in very young puppies ($n = 149$).

factor	AME	SE	z	p	Corrected	lower	upper
age	6.330	1.322	4.786	< 0.001	< 0.001	3.738	8.921
weight	1.504	0.314	4.786	< 0.001	< 0.001	0.888	2.120

This yields a interpretable regression equation of,

$$log(\text{GSH}_i) = 8.781 + 6.330\text{age}_i + 1.504\text{weight}_i + \epsilon_i.$$

To be interpretable in terms of the response variable, we solve this for GSH.

$$\text{GSH}_i = e^{8.781} \left(e^{6.330}\right)^{\text{age}_i} \left(e^{1.504}\right)^{\text{weight}_i} e^{\epsilon_i}.$$

The estimated coefficient of each variable intimates that an increase of one-unit of that explanatory variable would result in $(e^{\text{estimate}} - 1) \times 100$ percent change in the response. The reduced model shows that for all else held constant

- for every one month increase in age we can expect a $(e^{6.330} - 1) \times 100 = 56015.66\%$
- for every kg increase in weight we can expect a $(e^{1.504} - 1) \times 100 = 349.9652\%$

change in GSH (flu/20K cells), on average. These parameter estimates appear surprising but it is important to remember that the puppies in this subset of puppies range from a day old to just over a week old and all weigh less than 1kg.

For older dogs, none of the explanatory variables are considered significant.

Table H: The reduced model for GSH in older dogs ($n = 41$).

	Estimate	Std. Error	t value	$\Pr(> \lvert t \rvert)$
(Intercept)	10.317	0.174	59.24	< 0.001

This yields a regression equation of,

$$log(\text{GSH}_i) = 10.317 + \epsilon_i.$$

To be interpretable, in terms of the response variable, we solve this for GSH_i.

$$\text{GSH}_i = e^{10.317} e^{\epsilon_i} = 30242.39 \; e^{\epsilon_i}$$

3.2 Reactive Species (RS)

Table I: The reduced model for RS in puppies ($n = 159$).

	Estimate	Std. Error	t value	$\Pr(>\|t\|)$	Corrected
(Intercept)	10.607	0.099	107.395	< 0.001	< 0.001
sex=male	-0.382	0.132	-2.896	0.004	0.004

Table J: The reduced model for RS in very young puppies ($n = 149$).

	Estimate	Std. Error	t value	$\Pr(>\|t\|)$	Corrected
(Intercept)	10.618	0.101	105.028	< 0.001	< 0.001
sex=male	-0.365	0.138	-2.643	0.009	0.009

Table K: The reduced model for RS in older dogs ($n = 41$).

	Estimate	Std. Error	t value	$\Pr(>\|t\|)$	Corrected
(Intercept)	12.282	1.004	12.236	< 0.001	< 0.001
breed lifespan	-0.198	0.101	-1.954	0.058	0.058

3.3 Mitochondrial Content (MC)

Table L: The reduced model for MC in puppies ($n = 159$).

	Estimate	Std. Error	t value	$\Pr(>\|t\|)$
(Intercept)	10.430	0.094	110.600	< 0.001

Table M: The reduced model for MC in very young puppies ($n = 149$).

	Estimate	Std. Error	t value	Pr(> $\|t\|$)	Corrected
(Intercept)	9.836	0.215	45.692	< 0.001	< 0.001
age	5.935	1.952	3.040	0.003	0.003

Table N: The reduced model for MC in older dogs ($n = 41$).

	Estimate	Std. Error	t value	Pr(> $\|t\|$)
(Intercept)	10.638	0.156	68.340	< 0.001

3.4 Lipid Peroxidation Damage (LPO)

Table O: The reduced model for LPO in puppies ($n = 159$).

	Estimate	Std. Error	t value	Pr(> $\|t\|$)
(Intercept)	0.719	0.078	9.261	< 0.001

Table P: The reduced model for LPO in very young puppies ($n = 149$).

	Estimate	Std. Error	t value	Pr(> $\|t\|$)
(Intercept)	0.717	0.080	8.95	< 0.001

Table Q: The reduced model for LPO in older dogs ($n = 41$).

	Estimate	Std. Error	t value	Pr(> $\|t\|$)
(Intercept)	0.868	0.140	6.213	<0.001

3.5 DNA damage

Table R: The reduced model for DNA damage in puppies ($n = 39$).

	Estimate	Std. Error	t value	Pr($>|t|$)	Correct
(Intercept)	2.073	0.534	3.883	< 0.001	0.002
breed lifespan	-0.099	0.050	-1.958	0.058	0.058
source=ear clip	-1.028	0.310	-3.314	0.002	0.003
source=tail dock	-0.663	0.184	-3.614	0.001	0.002

Table S: The reduced model for DNA damage in very young puppies ($n = 36$).

	Estimate	Std. Error	t value	Pr($>|t|$)	Corrected
(Intercept)	2.090	0.555	3.766	0.001	0.002
breed lifespan	-0.100	0.052	-1.913	0.064	0.064
source=tail dock	-0.663	0.189	-3.511	0.001	0.002

Remark: Very young puppies only have samples from dew claws and tail docks.

Table T: The reduced model for DNA damage in older dogs ($n = 41$).

	Estimate	Std. Error	t value	Pr($>|t|$)
(Intercept)	0.465	0.076	6.089	<0.001

Note: we added one to the DNA damage measurements to avoid issues when performing the log transform of the response variable with measurements of zero.

3.6 Basal OCR

Table U: The reduced model for Basal OCR in puppies ($n = 159$).

	Estimate	Std. Error	t value	Pr($>|t|$)
(Intercept)	3.997	0.099	40.58	< 0.001

Table V: The reduced model for Basal OCR in very young puppies ($n = 149$).

	Estimate	Std. Error	t value	Pr(> |t|)	Corrected
(Intercept)	5.315	0.792	6.714	< 0.001	< 0.001
breed lifespan	-0.164	0.070	-2.361	0.020	0.020
weight	2.668	0.775	3.442	0.001	0.001
age × weight	-17.293	4.626	-3.739	< 0.001	0.001

Table W: The average marginal effects (AME) for Basal OCR in very young puppies ($n = 149$).

factor	AME	SE	z	p	Corrected	lower	upper
age	-7.196	1.925	-3.739	< 0.001	0.001	-10.969	-3.424
breed lifespan	-0.164	0.070	-2.361	0.018	0.027	-0.301	-0.028
weight	0.958	0.532	1.802	0.072	0.072	-0.084	2.000

Table X: The reduced model for Basal OCR in older dogs ($n = 41$).

	Estimate	Std. Error	t value	Pr(> |t|)	Corrected
(Intercept)	6.361	1.036	6.138	< 0.001	< 0.001
age	-0.215	0.086	-2.516	0.016	0.016

3.7 Proton leak

Table Y: The reduced model for proton leak in puppies ($n = 159$).

	Estimate	Std. Error	t value	Pr(> |t|)	Corrected
(Intercept)	5.717	0.666	8.583	0.001	0.001
breed lifespan	-0.296	0.066	-4.504	0.001	0.001

Table Z: The reduced model for proton leak in very young puppies ($n = 149$).

	Estimate	Std. Error	t value	Pr(> |t|)	Corrected
(Intercept)	5.761	0.682	8.446	0.001	0.001
breed lifespan	-0.298	0.067	-4.442	0.001	0.001

Table Aa: The reduced model for proton leak in older dogs ($n = 41$).

	Estimate	Std. Error	t value	Pr(> $\|t\|$)	Corrected
(Intercept)	1.819	0.521	3.491	0.001	0.002
weight	0.039	0.019	2.033	0.049	0.049

3.8 ATP Production

Table Ab: The reduced model for ATP in puppies ($n = 159$).

	Estimate	Std. Error	t value	Pr(> $\|t\|$)
(Intercept)	3.475	0.118	29.56	< 0.001

Table Ac: The reduced model for ATP in very young puppies ($n = 149$).

	Estimate	Std. Error	t value	Pr(> $\|t\|$)	Corrected
(Intercept)	4.301	0.268	16.069	< 0.001	< 0.001
age	-7.800	2.427	-3.214	0.002	0.002

Table Ad: The reduced model for ATP in older dogs ($n = 41$).

	Estimate	Std. Error	t value	Pr(> $\|t\|$)	Corrected
(Intercept)	5.233	0.944	5.541	< 0.001	< 0.001
age	-0.165	0.078	-2.119	0.04	0.04

3.9 Spare Respiratory Capacity

Table Ae: The reduced model for spare respiratory capacity in puppies ($n = 159$).

	Estimate	Std. Error	t value	Pr(> $\|t\|$)
(Intercept)	3.831	0.312	12.274	< 0.001
breed lifespan	0.088	0.031	2.858	0.005

Table Af: The reduced model for spare respiratory capacity in very young puppies ($n = 149$).

	Estimate	Std. Error	t value	Pr(> |t|)	Corrected
(Intercept)	3.766	0.320	11.753	< 0.001	< 0.001
breed lifespan	0.093	0.032	2.946	0.004	0.004

Table Ag: The reduced model for spare respiratory capacity in older dogs ($n = 41$).

	Estimate	Std. Error	t value	Pr(> |t|)
(Intercept)	4.720	0.056	83.67	< 0.001

3.10 Coupling Efficiency

Note: A log transform was not required for the coupling efficiency response - these parameter estimates should be interpreted as they would for a usual linear model.

Table Ah: The reduced model for coupling efficiency in puppies ($n = 158$).

	Estimate	Std. Error	t value	Pr(> |t|)	Corrected
(Intercept)	20.577	13.937	1.476	0.142	0.142
age	-9.330	3.768	-2.476	0.014	0.022
breed lifespan	4.735	1.368	3.461	0.001	0.002

Table Ai: The reduced model for coupling efficiency in very young puppies ($n = 148$).

	Estimate	Std. Error	t value	Pr(> |t|)	Corrected
(Intercept)	35.639	14.415	2.472	0.015	0.015
age	-133.694	41.128	-3.251	0.001	0.002
breed lifespan	4.447	1.345	3.306	0.001	0.002

Table Aj: The reduced model for coupling efficiency in older dogs ($n = 41$).

	Estimate	Std. Error	t value	Pr(> |t|)
(Intercept)	61.43	4.14	14.84	< 0.001

3.11 Total Glycolysis

Table Ak: The reduced model for total glycolysis in puppies ($n = 159$).

	Estimate	Std. Error	t value	$Pr(>\|t\|)$	Corrected
(Intercept)	5.696	0.119	47.923	< 0.001	< 0.001
source=ear clip	-1.226	0.326	-3.765	< 0.001	< 0.001
source=tail dock	-1.099	0.158	-6.942	< 0.001	< 0.001

Table Al: The reduced model for total glycolysis in very young puppies ($n = 149$).

	Estimate	Std. Error	t value	$Pr(>\|t\|)$	Corrected
(Intercept)	5.696	0.119	47.743	< 0.001	< 0.001
source=tail dock	-1.099	0.159	-6.916	< 0.001	< 0.001

Remark: Very young puppies only have samples from dew claws and tail docks.

Table Am: The reduced model for total glycolysis in older dogs ($n = 41$).

	Estimate	Std. Error	t value	$Pr(>\|t\|)$
(Intercept)	4.413	0.190	23.21	< 0.001

3.12 Total Glycolytic Capacity

Table An: The reduced model for total glycolytic capacity in puppies ($n = 159$).

	Estimate	Std. Error	t value	$Pr(>\|t\|)$	Corrected
(Intercept)	5.719	0.122	46.899	< 0.001	< 0.001
size=small	-0.576	0.177	-3.245	0.001	0.001
source=ear clip	-1.331	0.327	-4.075	< 0.001	< 0.001
source=tail dock	-0.876	0.166	-5.268	< 0.001	< 0.001

Table Ao: The reduced model for total glycolytic capacity in very young puppies ($n = 149$).

	Estimate	Std. Error	t value	Pr($>|t|$)	Corrected
(Intercept)	5.719	0.122	46.804	< 0.001	< 0.001
source=tail dock	-0.876	0.167	-5.257	< 0.001	< 0.001
size=small	-0.576	0.178	-3.239	0.001	0.001

Remark: Very young puppies only have samples from dew claws and tail docks.

Table Ap: The reduced model for total glycolytic capacity in older dogs ($n = 41$).

	Estimate	Std. Error	t value	Pr($>|t|$)	Corrected
(Intercept)	7.523	1.440	5.225	< 0.001	< 0.001
breed lifespan	-0.318	0.146	-2.187	0.035	0.035

3.13 Non-Glycolytic Acidification

Table Aq: The reduced model for non-glycolytic acidification in puppies ($n = 159$).

	Estimate	Std. Error	t value	Pr($>|t|$)	Corrected
(Intercept)	6.359	0.561	11.329	< 0.001	< 0.001
size=small	-0.497	0.189	-2.634	0.009	0.009
source=ear clip	-0.968	0.338	-2.859	0.005	0.007
source=tail dock	-0.486	0.173	-2.816	0.005	0.007
breed lifespan	-0.191	0.055	-3.451	0.001	0.002

Table Ar: The reduced model for non-glycolytic acidification in very young puppies ($n = 149$).

	Estimate	Std. Error	t value	Pr($>|t|$)	Corrected
(Intercept)	5.984	0.577	10.370	< 0.001	< 0.001
size	-0.667	0.186	-3.577	< 0.001	0.001
breed lifespan	-0.175	0.058	-3.031	0.003	0.003

Table As: The reduced model for non-glycolytic acidification in older dogs ($n = 41$).

	Estimate	Std. Error	t value	Pr(> $\|t\|$)	Corrected
(Intercept)	7.139	1.782	4.005	< 0.001	0.001
breed lifespan	-0.370	0.180	-2.055	0.047	0.047

References

Benjamini, Y. and Hochberg, Y. (1995). Controlling the False Discovery Rate: A Practical and Powerful Approach to Multiple Testing. *Journal of the Royal Statistical Society. Series B (Methodological)*, 57(1):289–300.

Dunn, O. J. (1961). Multiple comparisons among means. *Journal of the American Statistical Association*, 56(293):52–64.

Leeper, T. J. (2017). *margins: Marginal Effects for Model Objects*. R package version 0.3.0.

McLeod, A. and Xu, C. (2017). *bestglm: Best Subset GLM*. R package version 0.36.

Morgan, J. and Tatar, J. (1972). Calculation of the residual sum of squares for all possible regressions. *Technometrics*, 14:317–325.

R Core Team (2016). *R: A Language and Environment for Statistical Computing*. R Foundation for Statistical Computing, Vienna, Austria.

Schwarz, G. et al. (1978). Estimating the dimension of a model. *The annals of statistics*, 6(2):461–464.
